# Post‐translational control of ABA signalling: the roles of protein phosphorylation and ubiquitination

**DOI:** 10.1111/pbi.12652

**Published:** 2016-12-06

**Authors:** Wenqi Yang, Wei Zhang, Xiaoxue Wang

**Affiliations:** ^1^ Rice Research Institute Shenyang Agricultural University Shenyang China

**Keywords:** abscisic acid, ABA signalling, post‐translational regulation, phosphorylation, dephosphorylation, ubiquitination, 26S proteasome system

## Abstract

The plant phytohormone abscisic acid (ABA) plays significant roles in integrating environmental signals with embryogenesis, germination, seedling establishment, the floral transition and the adaptation of plants to stressful environments by modulating stomatal movement and stress‐responsive gene expression. ABA signalling consists of ABA perception, signal transduction and ABA‐induced responses. ABA receptors such as members of the PYR/PYL family, group A type 2C protein phosphatases (as negative regulators), SnRK2 protein kinases (as positive regulators), bZIP transcription factors and ion channels are key components of ABA signalling. Post‐translational modifications, including dephosphorylation, phosphorylation and ubiquitination, play important roles in regulating ABA signalling. In this review, we focus on the roles of post‐translational modifications in ABA signalling. The studies presented provide a detailed picture of the ABA signalling network.

## Introduction

As sessile organisms, plants must adapt to their environment to survive, develop and propagate‐especially under stressful conditions. The phytohormone abscisic acid (ABA) is a key regulator of plant growth and development and of the responses of plants to biotic and abiotic stresses (Hirayama and Shinozaki, 2007; Melotto *et al*., 2006). ABA is involved in promoting embryo maturation, initiating and maintaining seed dormancy, repressing seed germination and postgermination growth, inhibiting the transition from vegetative to reproductive growth, modulating stomatal movement, inducing gene expression and improving plant adaptation to stressful environments (Cutler *et al*., 2010; Finkelstein, 2013). Much work has been carried out to discover the mechanisms underlying ABA signalling given the hormone's significance in plant development and its potential agricultural applications, and significant progress has been made. Five core components are involved in ABA signalling: ABA receptors, negative regulators, positive regulators, ABA‐responsive transcription factors and ABA‐responsive genes (Hauser *et al*., 2011).

The identification of intracellular ABA receptors, including those belonging to the pyrabactin resistance 1 (PYR1)/PYR1‐like (PYLs)/regulatory components of ABA receptors (RCAR) family (hereafter referred to as PYR/PYLs), has greatly enhanced our understanding of ABA signalling (Ma *et al*., 2009; Park *et al*., 2009; Santiago *et al*., 2009). PYR/PYLs belong to the steroidogenic acute regulatory protein‐related lipid transfer domain protein family, which is also called the Bet v 1 superfamily (Iyer *et al*., 2001; van Loon *et al*., 2006; Radauer *et al*., 2008). In *Arabidopsis*, fourteen PYR/PYLs have been identified (PYR1 and PYL1–PYL13), which are localized to the cytoplasm, plasma membrane or nucleus (McConnell *et al*., 2001; Park *et al*., 2009) (Table 1).

**Table 1:** PYR/PYL ABA receptor and PP2C interactions identified in *Arabidopsis*

ABA receptor	AGI annotation	PP2Cs interacted with	References
PYR1/RCAR11	AT4G17870	ABI1, ABI2, HAB1, HAB2, PP2CA	Fujii *et al*. (2009), Hao *et al*. (2011), Park *et al*. (2009)
PYL1/RCAR12	AT5G46790	ABI1, ABI2, HAB1, HAB2, PP2CA	Fujii *et al*. (2009), Hao *et al*. (2011), Ma *et al*. (2009), Park *et al*. (2009)
PYL2/RCAR14	AT2G26040	ABI1, HAB1, HAB2, PP2CA	(Fujii *et al*., 2009; Hao *et al*., 2011; Park *et al*., 2009)
PYL3/RCAR13	AT1G73000	ABI1, HAB1, HAB2, PP2CA	Fujii *et al*. (2009), Hao *et al*. (2011), Park *et al*. (2009)
PYL4/RCAR10	AT2G38310	ABI1, HAB1, HAB2, PP2CA	Fujii *et al*. (2009), Hao *et al*. (2011), Park *et al*. (2009), Pizzio *et al*. (2013)
PYL5/RCAR8	AT5G05440	ABI1, ABI2, HAB1, HAB2, PP2CA	Fujii *et al*. (2009), Hao *et al*. (2011), Ma *et al*. (2009), Santiago *et al*. (2009)
PYL6/RCAR9	AT2G40330	ABI1, HAB1, HAB2, PP2CA	Fujii *et al*. (2009), Hao *et al*. (2011), Santiago *et al*. (2009)
PYL7/RCAR2	AT4G01026	ABI1	Fujii *et al*. (2009)
PYL8/RCAR3	AT5G53160	ABI1, ABI2, HAB1, HAB2, PP2CA	Fujii *et al*. (2009), Hao *et al*. (2011), Ma *et al*. (2009), Santiago *et al*. (2009)
PYL9/RCAR1	AT1G01360	ABI1, ABI2, HAB1, HAB2, PP2CA	Fujii *et al*. (2009), Hao *et al*. (2011), Ma *et al*. (2009), Park *et al*. (2009)
PYL10/RCAR4	AT4G27920	ABI1, HAB1, HAB2, PP2CA	Fujii *et al*. (2009), Hao *et al*. (2011)
PYL11/RCAR5	AT5G45860	ABI1	Fujii *et al*. (2009)
PYL12/RCAR6	AT5G45870	PP2CA/AHG3	Fujii *et al*. (2009), Park *et al*. (2009)
PYL13/RCAR7	AT4G18620	PP2CA/AHG3, ABI1, ABI2	Fuchs *et al*. (2014), Zhao *et al*. (2013)

**Figure 1:**
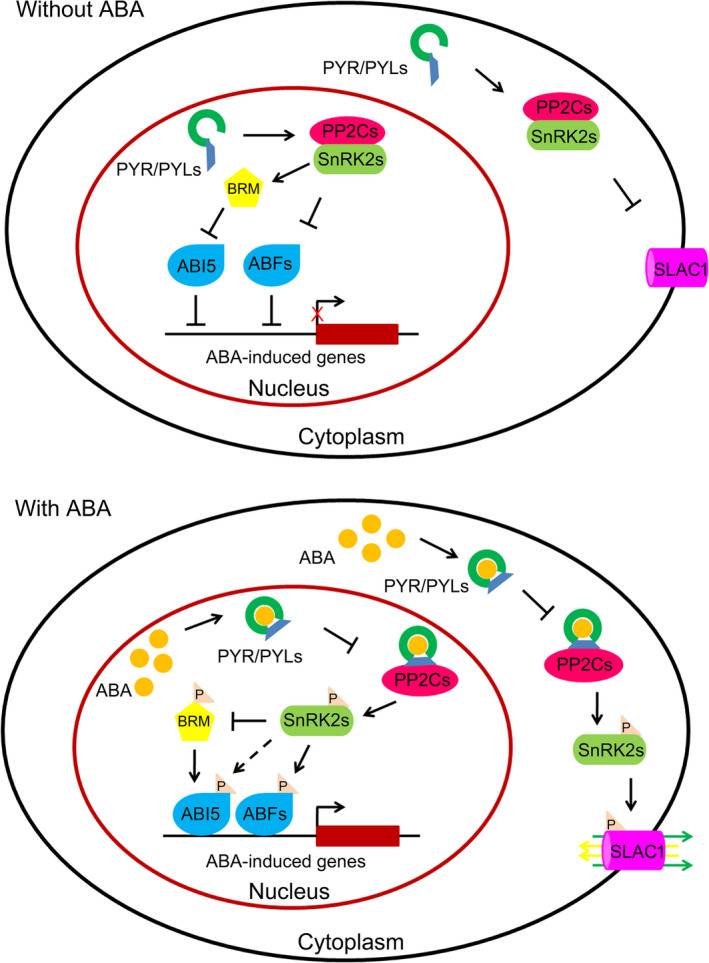
The ABA signalling cascade in *Arabidopsis*. The components involved in ABA signalling include the PYR/PYL family of ABA receptors, PP2Cs (negative regulator), SnRK2s (positive regulator), SWI/SNF chromatin‐remodelling ATPase BRM, transcription factors (AREB/ABFs, ABI5), ABA‐responsive genes and S‐type anion channels. ABA signalling occurs in the cytoplasm, nucleus and plasma membrane. In the presence of ABA, a complex is formed between ABA, receptor proteins and PP2Cs (ABA‐PYR/PYL/RCAR‐PP2C), which prevents PP2Cs from inhibiting the phosphorylation activity of SnRK2s. SnRK2s, phosphorylated via an unknown mechanism, then activate bZIP transcription factors and S‐type anion channels (e.g. SLAC1) and inactivate SWI/SNF chromatin‐remodelling ATPase BRM by phosphorylation. Once activated or inactivated, these proteins trigger the expression of ABA‐responsive genes or regulate the exchange of ions to confer plant tolerance to abiotic stresses.

The dephosphorylation activity of group A protein phosphatase type 2Cs (PP2Cs) and the phosphorylation activity of sucrose nonfermenting 1 (SNF1)‐related protein kinases 2 (SnRK2s) are required for ABA signalling (Ma *et al*., 2009; Park *et al*., 2009; Santiago *et al*., 2009). In the absence of ABA, a physical association exists between PP2Cs and C‐terminal subdomain II of SnRK2s; this inhibits the phosphorylation activity of SnRK2s and blocks ABA signal transduction. PP2Cs repress ABA signalling by dephosphorylating and inactivating SnRK2s (Fujii *et al*., 2007; Mustilli *et al*., 2002; Umezawa *et al*., 2009; Vlad *et al*., 2009; Yoshida *et al*., 2006a) (Figure 1). Following the induction of ABA synthesis by environmental or developmental factors, the hormone is perceived and bound by PYR/PYL family ABA receptors (Ma *et al*., 2009; Park *et al*., 2009; Santiago *et al*., 2009). The binding of ABA results in conformational changes in the receptor proteins, generating a platform for interaction with PP2Cs. The interaction between PYR/PYLs and PP2Cs suppresses the phosphatase activity of the enzymes and relieves the inhibition of SnRK2s (Nishimura *et al*., 2010; Park *et al*., 2009; Soon *et al*., 2012; Yin *et al*., 2009) (Figure 1). The released SnRK2s are then activated by autophosphorylation or phosphorylation via other kinases (Fujii *et al*., 2009; Ng *et al*., 2011; Soon *et al*., 2012). These activated SnRK2s are able to phosphorylate downstream proteins or transcription factors, including basic leucine zipper (bZIP) transcription factors called ABA‐responsive element binding factors (AREBs/ABFs) and S‐type anion channels (e.g. slow anion channel 1, SLAC1), to induce ABA responses (Brandt *et al*., 2012; Fujii *et al*., 2009; Geiger *et al*., 2009; Umezawa *et al*., 2009; Xue *et al*., 2011) (Figure 1).

In addition to dephosphorylation and phosphorylation, polyubiquitination mediated by E3 ubiquitin (Ub) ligases has been found to be important in ABA perception, ABA signal transduction and the induction of ABA responses. PYR/PRLs, PP2Cs, type 2A protein phosphatases (PP2As), transcription factors and proteins encoded by ABA‐responsive genes are targeted for E3 Ub ligase‐mediated polyubiquitination and degraded by the 26S proteasome. Several types of E3 Ub ligases are involved in ABA signalling. In this review, we focus on recent discoveries related to the post‐translational regulation of ABA signalling.

## Dephosphorylation negatively regulates ABA signalling

PP2Cs and PP2As are negative regulators of ABA signalling. There are 76 PP2Cs in *Arabidopsis*, which are clustered into 10 groups (Fuchs *et al*., 2013). Nine PP2Cs involved in ABA signalling belong to group A: ABA‐insensitive (ABI) 1, ABI2, ABA‐hypersensitive germination (AHG) 1, AHG3/PP2CA, hypersensitive to ABA (HAB) 1, HAB2, highly ABA‐induced (HAI) 1, AKT1‐interacting PP2C 1 (AIP1)/HAI2 and HAI3 (Kuhn *et al*., 2006; Merlot *et al*., 2001; Robert *et al*., 2006; Rubio *et al*., 2009; Saez *et al*., 2004, 2006; Yoshida *et al*., 2006b). A common feature of clade A PP2Cs is that their expression is induced by high ABA levels or stressful conditions (Fujita *et al*., 2009, 2011). Loss‐of‐function mutations in genes encoding clade A PP2Cs cause ABA hypersensitivity, except for *HAI1*,* AIP1/HAI2* and *HAI3* (which encode the so‐called HAI PP2Cs) (Gosti *et al*., 1999; Merlot *et al*., 2001; Nishimura *et al*., 2007; Yoshida *et al*., 2006b). Single mutations in genes encoding HAI PP2Cs were not found to produce ABA‐responsive phenotypes (Yoshida *et al*., 2006b). The *hai* double or triple mutants show ABA‐hyposensitive phenotypes in seed germination, which contrast with the hypersensitive phenotypes of other group A PP2C mutants, and ABA‐hypersensitive responses in postgermination growth, which is similar to other group A PP2C mutants (Bhaskara *et al*., 2012). Furthermore, *hai1‐1* mutation elevates the ABA response of *pp2ca‐1/ahg3‐1* mutant. The *pp2ca‐1hai1‐1* (or *ahg3‐1hai1‐1*) double mutant plants exhibit enhanced ABA‐mediated growth inhibition, increased ABA‐responsive gene induction and diminished water loss (Antoni *et al*., 2012). However, *hai* single mutant exhibits enhanced drought tolerance phenotypes, including the accumulation of proline and osmoregulatory solute and increased expression of abiotic stress‐responsive genes, which is not observed in other group A PP2C mutants. These results suggest that HAI PP2Cs have a greater role in ABA‐independent signalling than in ABA‐dependent signalling in response to drought stress (Bhaskara *et al*., 2012).

The selective inhibition of PP2Cs by a complex composed of PRY/PYLs and ABA has been reported (Antoni *et al*., 2012; Bhaskara *et al*., 2012) (Table 1). In the absence of ABA, clade A PP2Cs bind directly to and dephosphorylate SnRK2s, suppressing their kinase activity and blocking ABA signalling (Soon *et al*., 2012; Umezawa *et al*., 2009; Vlad *et al*., 2009). These results reveal the negative regulatory roles and functional differentiation of PP2Cs in the perception and transduction of ABA signals (Bhaskara *et al*., 2012; Cutler *et al*., 2010).

**Figure 2:**
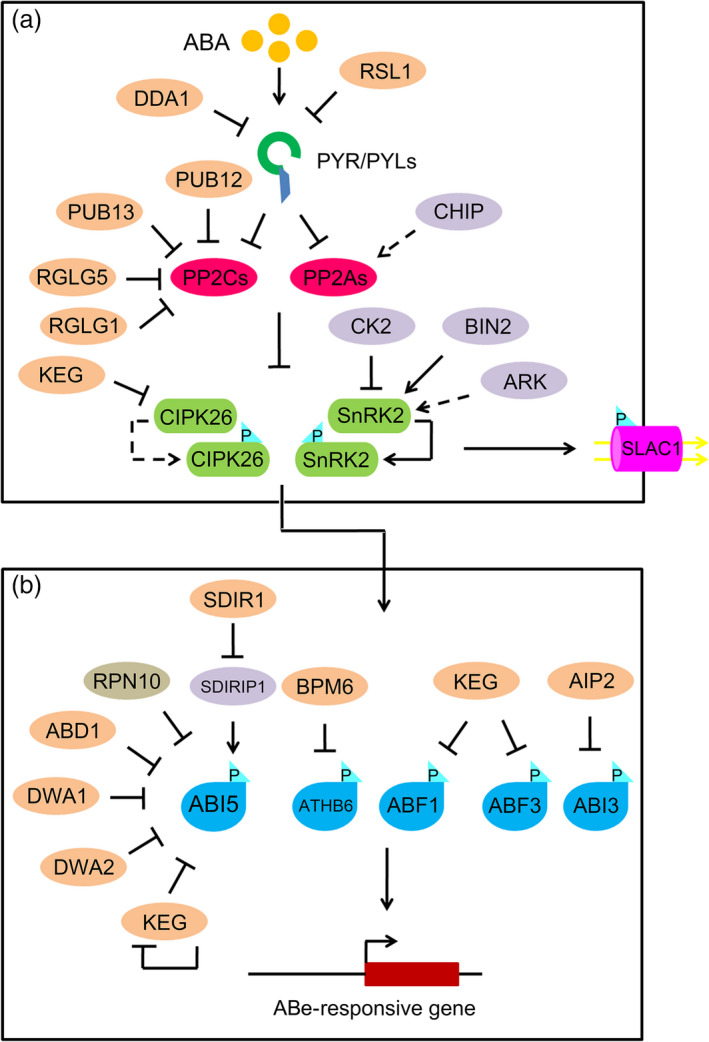
The regulatory roles of protein phosphorylation and ubiquitination in ABA signalling. (a) Post‐translational regulation of PYR/PYL ABA receptors, PP2Cs and PP2As phosphatases, SnRK2s and CIPK26 protein kinases in ABA signalling. DDA1 and RSL1, Cul4‐based and RING‐type E3 Ub ligases, are able to target PYR/PYL ABA receptors for degradation by the Ub‐mediated 26S proteasome pathway. Two U‐box E3 ligases (e.g. PUB12 and PUB13) and two RING E3 ligases (e.g. RGLG1 and RGLG5) target PP2Cs for degradation by the Ub‐mediated 26S proteasome pathway. Similar to the functions of PP2Cs, PP2AAs and PP2ABs regulatory subunits in PP2A complexes are negatively involved in ABA signalling. CHIP, a U‐box E3 ligase, uses it monoubiquitination activity to control the expression of RCN1 and PP2AA3 regulatory subunits in PP2A complexes. SnRK2s are able to be phosphorylated and activated by themselves, or by BIN2 or ARK to activate downstream ABA signalling. In contrast, OST1, a SnRK2 kinase involved in ABA signalling, is phosphorylated and inactivated by CK2 kinase to block downstream ABA signalling. CIPK26, a novel protein kinase, interacts with PP2Cs in the absence of ABA and phosphorylates downstream transcription factors such as ABI5 to trigger ABA responses. KEG, a RING‐type E3 ligase, can target CIPK26 for disruption. (b) Downstream transcription factors (e.g. ABI5, ABI3, ABFs and ATHB6) and ion channels are the substrates of SnRK2s or CIPK26, which are also the targets of E3 ligases. ABD1, DWA1 and DWA2, as Cul4‐based E3 ligases, interact with and ubiquitinate ABI5 for degradation by the 26 proteasome. RPN10, a core component of the 26S proteasome, targets ABI5 for degradation. In addition to targeting CIPK26 for degradation, KEG can target several substrates for disruption, including ABI5, ABF1 and ABF3. The self‐ubiquitination of KEG is triggered by a high level of ABA, causing the degradation of KEG and releasing ABI5. SDIR1, a RING finger E3 ligase, targets SDIRIP1 for degradation to regulate ABI5. In addition, AIP2 and BPM6, RING‐type and CUL3‐based E3 ligases, interact with and phosphorylate ABI3 and ATHB6, respectively, causing their degradation. Black arrows represent promotion. Black T‐shaped bars represent repression. Dashed lines represent unconfirmed relationships. P, phosphate.

Recently, PP2As were shown to interact with SnRK2s and negatively regulate ABA signalling (Waadt *et al*., 2015) (Figure 2). PP2As are heterotrimeric holoenzyme complexes consisting of the following subunits: PP2AA, PP2AB and PP2AC (Shi, 2009; Xu *et al*., 2006). There are five catalytic subunits (PP2AC), three PP2AA‐regulatory subunits and 18 PP2AB‐regulatory subunits in *Arabidopsis* genome (DeLong, 2006; Farkas *et al*., 2007). The B subunits are classified into four subtypes, which comprise four subtypes: 2 B, 9 B′, 6 B″ and 1 TAP46 (Farkas *et al*., 2007). Three PP2A subunits, including PP2AA1/roots curl in NPA 1 (RCN1), PP2AA2 and PP2AA3, form ternary complexes to conduct their functions. Both PP2AAs and PP2ABs function as regulatory factors, whereas PP2ACs act as catalytic components of PP2A ternary complexes (DeLong, 2006; Farkas *et al*., 2007).

Genetic studies have revealed the complicated roles of PP2As in ABA signalling (Charpentier *et al*., 2014; Kwak *et al*., 2002; Pernas *et al*., 2007; Saito *et al*., 2008). The regulatory PP2AA and PP2AB' subunits regulate ABA signalling through interacting with a SnRK2, open stomata 1 (OST1). The *rcn1* mutant and *pp2aa*‐regulatory subunit double mutants exhibit ABA insensitivity during seed germination and stomatal closure (Kwak *et al*., 2002; Saito *et al*., 2008; Waadt *et al*., 2015). Further, the mutations *pp2ac3*,* pp2ac4* and *pp2ac5* confer ABA‐insensitive phenotypes in seed germination (Waadt *et al*., 2015). This suggests that PP2AA, PP2AB′, and PP2AC3, PP2AC4, PP2AC5 subunits might positively regulate ABA signalling in seed germination and stomatal closure. In contrast, the catalytic subunit mutant *pp2ac2* shows ABA hypersensitivity during seed germination, root growth and seedling development (Pernas *et al*., 2007). These results suggest that the roles of PP2A regulatory and catalytic subunits in ABA response and ABA signalling might be different (Waadt *et al*., 2015). The exact mechanisms underlying the regulation of ABA signalling by PP2As and the relationship between clade A PP2Cs and PP2As are unclear.

## Phosphorylation positively regulates ABA signalling

Several SnRK2s in *Arabidopsis* have been identified as ABA‐ or stress‐activated kinases (Mustilli *et al*., 2002; Yoshida *et al*., 2002). Specifically, there are 38 SnRKs, 10 of which are subgroup III SnRKs, or SnRK2s (Hrabak *et al*., 2003). These SnRK2s, including SnRK2.2/SRK2D, SnRK2.3/SRK2I and SnRK2.6/SRK2E/OST1, are activated by ABA in *Arabidopsis* protoplasts and perform as positive regulators of ABA signalling (Boudsocq *et al*., 2004, 2007; Fujii *et al*., 2007; Mustilli *et al*., 2002). Genomewide data indicate that the expression of a vast number of ABA‐ and osmotic stress‐responsive genes is impaired in the *snrk2.2/snrk2.3/snrk2.6* triple mutant and the snrk2.2/2.3/2.6 triple mutant is practically bind to ABA, suggesting a positive role for SnRK2s in ABA signalling (Fujii and Zhu, 2009; Fujita *et al*., 2009). The phosphorylation activities of SnRK2s are suppressed via a physical association with PP2Cs in the absence of ABA (Soon *et al*., 2012; Umezawa *et al*., 2009; Vlad *et al*., 2009). However, in the presence of ABA, PYR/PYLs bind to the hormone and cause conformation changes to interact with PP2Cs; this interaction represses PP2C activity and releases SnRK2 suppression, triggering downstream ABA responses (Ma *et al*., 2009; Melcher *et al*., 2009; Miyazono *et al*., 2009; Nishimura *et al*., 2009; Park *et al*., 2009).

SnRK2s regulate a wide range of proteins through ABA‐mediated phosphorylation. AREB/ABFs, ABI3 and ABI5 are ABA response‐related transcription factors and targets of SnRK2s (Furihata *et al*., 2006; Kobayashi *et al*., 2005; Sirichandra *et al*., 2010). SnRK2s can also phosphorylate plasma membrane proteins. For example, the guard cell kinase SnRK2.6/OST1 has been shown to phosphorylate ion channels such as SLAC1 (Brandt *et al*., 2012; Geiger *et al*., 2009; Lee *et al*., 2009), quick anion channel 1/aluminium‐activated malate transporter 12 (Imes *et al*., 2013; Sasaki *et al*., 2010), the potassium channel KAT1 (Sato *et al*., 2009), NADPH oxidase respiratory burst oxidase homolog F (Sirichandra *et al*., 2009a) and the anion/proton exchanger CLCa (Wege *et al*., 2014), which play important roles in controlling stomatal aperture. Recently, SWI/SNF chromatin‐remodelling ATPase BRAHMA (BRM) in *Arabidopsis* was identified as the target protein of SnRK2s (Peirats‐Llobet *et al*., 2016). BRM is inactivated by SnRK2‐dependent phosphorylation and activated by PP2C‐dependent dephosphorylation. Phosphorylation of BRM by SnRK2s inhibits the activity of BRM, releasing the expression of *ABI5*. The *brm‐3* mutant exhibits ABA‐hypersensitive phenotype through derepressing *ABI5* (Han *et al*., 2012; Peirats‐Llobet *et al*., 2016). These findings suggest that BRM acts as a negative regulator of ABA signalling and connect ABA signalling to chromatin remodelling (Han *et al*., 2012; Peirats‐Llobet *et al*., 2016) (Figure 1). In addition, putative substrates have been identified through the mapping of peptide phosphorylation preferences and phosphoproteomic approaches (Shin *et al*., 2007; Sirichandra *et al*., 2010; Umezawa *et al*., 2013; Vlad *et al*., 2008; Wang *et al*., 2013). However, the functions of these putative substrates in ABA responses require validation.

In addition to SnRK2s, calcineurin B‐like interacting protein kinase 26 (CIPK26) has been found to interact with ABI1 and ABI2 during ABA signalling (Lyzenga *et al*., 2013) (Figure 2). CIPK26 also interacts with and phosphorylates ABI5 to activate downstream ABA responses. *CIPK26* overexpression produces a hypersensitive phenotype in seed germination, suggesting a positive role for CIPK26 in ABA signalling (Lyzenga *et al*., 2013). Thus, CIPK26 regulates ABA signalling by phosphorylating and activating ABI5 (Lyzenga *et al*., 2013).

The regulation of SnRK2s has also been studied, and SnRK2 activity has been shown to be controlled by phosphorylation. The autophosphorylation activity levels of recombinant SnRK2.2 and SnRK2.3 were weaker than those of SnRK2.6, suggesting that some SnRK2s are activated by as yet unknown kinases *in vivo* (Ng *et al*., 2011). Recently, brassinosteroid (BR)‐insensitive 2 (BIN2), casein kinase 2 (CK2), and ABA and abiotic stress‐responsive Raf‐like kinase (ARK) were identified as kinases that regulate SnRK2 activity and stability (Cai *et al*., 2014; Saruhashi *et al*., 2015; Vilela *et al*., 2015).

BIN2 is a glycogen synthase kinase 3 (GSK3)‐like kinase that can phosphorylate and regulate the activity, stability and subcellular localization of a number of proteins in diverse systems (Grimes and Jope, 2001; Saidi *et al*., 2012). Previous studies showed that BIN2 functions as a negative regulator of BR signalling by phosphorylating transcription factors, including brassinazole‐resistant 1 and BRI1‐EMS‐suppressor 1 (He *et al*., 2002; Yin *et al*., 2002). In the ABA signalling pathway, BIN2 phosphorylates SnRK2.2 and SnRK2.3, enhancing their kinase activity levels. These data have provided significant insight into the modulation of ABA signalling by GSK3‐like kinases and crosstalk between BR signalling and ABA signalling in *Arabidopsis* (Cai *et al*., 2014; Yan *et al*., 2009) (Figure 2). Recently, ARK was identified as a novel regulatory component of SnRK2s in *Physcomitrella patens* (Saruhashi *et al*., 2015) (Figure 2). ARK is a group B3 Raf‐like MAP kinase kinase kinase (B3‐MAPKKK). A moss *ark* mutant showed reduced ABA sensitivity and reduced tolerance to hyperosmosis (Saruhashi *et al*., 2015). The mutant also showed impaired phosphorylation activity by SnRK2s, indicating that ARK is an essential signalling component regulating SnRK2 activity in basal land plants such as moss (Saruhashi *et al*., 2015). Six B3‐MAPKKKs are encoded in the *Arabidopsis* genome. *B3‐MAPKKK* genes from *Arabidopsis* can recover the ABA‐insensitive phenotype of the moss *ark* mutant, indicating that the functions of B3‐MAPKKKs in ABA signalling in *Arabidopsis* are similar to those in moss (Saruhashi *et al*., 2015). However, the functions of *Arabidopsis* B3‐MAPKKKs in ABA signalling are unclear. In addition, CK2 has been characterized as a regulator of SnRK2.6/SRK2E/OST1 protein stability (Vilela *et al*., 2015). CK2 is a highly conserved serine/threonine kinase with multiple subunits in eukaryotes (Meggio and Pinna, 2003). The CK2 holoenzyme has two catalytic ɑ‐subunits and two regulatory β‐subunits. There are four *ɑ‐* and four β‐subunits in both the *Arabidopsis* and maize genomes (Riera *et al*., 2011; Salinas *et al*., 2006); CK2 phosphorylates ZmOST1 *in planta* to increase its binding to PP2C; this, in turn, triggers ZmOST1 protein degradation and affects its kinase activity (Vilela *et al*., 2015). These results indicate that CK2 is a negative regulator of ABA signalling and link CK2 directly to the core ABA signalling pathway (Vilela *et al*., 2015). However, *ck2a* mutants are hyposensitive to ABA during seed germination, cotyledon greening and stomatal opening due to the down‐regulated expression of *OST1* and other ABA‐responsive genes (Mulekar *et al*., 2012; Wang *et al*., 2014). These contradictory results indicate that CK2 might have additional functions in regulating ABA signalling.

SnRK2s are key components of ABA signalling cascades. Understanding their activation, degradation mechanisms and substrates will increase our understanding of ABA signalling.

## Ub‐mediated protein degradation regulates ABA signalling

Ubiquitination, a type of protein post‐translational modification, is involved in many aspects of plant development and environmental responses. Three enzymes are involved in ubiquitination: E1, as a Ub‐activating enzyme; E2, as a Ub‐conjugating enzyme; and E3, as a Ub‐protein ligase (Smalle and Vierstra, 2004). Upon polyubiquitination, substrate proteins undergo proteolysis by the 26S proteasome. Ub‐mediated protein degradation via the 26S proteasome thus provides a master mechanism for controlling the abundance of key regulators in *Arabidopsis*. E3 Ub ligases are responsible for the specificity of the system by interacting with the substrate; substrate recognition enables the transfer of the 76‐amino acid Ub molecule to a lysine residue within the target protein (Smalle and Vierstra, 2004). E3 Ub ligases are classified into four types: really interesting new gene (RING), cullin‐RING ligase (CRLs), U‐box and homologous to E6‐AP carboxyl terminus (Vierstra, 2009). The CRLs can be further divided into four subtypes: anaphase‐promoting complex, S‐phase kinase‐associated protein 1‐cullin 1‐F‐box, DNA damage‐binding protein (DDB) and bric‐a‐brac/tramtrack/broad complex (BTB) (Hua and Vierstra, 2011). Recently, CRLs, RING and U‐box E3 Ub ligases were found to be involved in ABA signalling (Yu *et al*., 2015) (Table 2 and Figure 2).

**Table 2:** The roles of E3 ubiquitin ligases and the 26S proteasome in ABA signalling

Type of E3 ligase	Name	Effect on ABA signalling	Target	References
CUL4‐DDB1	DDA1	Negative^a^	PYL4, PYL8, PYL9	Irigoyen *et al*. (2014)
	DWA1	Negative	ABI5	Lee *et al*. (2010)
	DWA2	Negative	ABI5	Lee *et al*. (2010)
	ABD1	Negative	ABI5	Seo *et al*. (2014)
CUL3‐BTB	BPM6	Negative	ATHB6	Lechner *et al*. (2011)
RING	RSL1	Negative	PYR1, PYL4	Bueso *et al*. (2014)
	KEG	Negative	CIPK26, ABI5, ABF1, ABF3	Chen *et al*. (2013), Liu and Stone (2010, 2013), Lyzenga *et al*. (2013), Stone *et al*. (2006)
	SDIR1	Positive^b^	SDIRIP1	Zhang *et al*. (2015, 2007)
	AIP2	Negative	ABI3	Kurup *et al*. (2000)
	AIRP3	Positive	RD21	Kim and Kim (2013), Pratelli *et al*. (2012)
U‐box	PUB12	Positive	ABI1	Kong *et al*. (2015)
	PUB13	Positive	ABI1	Kong *et al*. (2015)
	CHIP	Positive	PP2A	Luo *et al*. (2006)
26S proteasome	RPN10	Negative	ABI5	Smalle *et al*. (2003)

a Negative: inhibition of ABA signalling.

b Positive: activation of ABA signalling.

### Two E3 ligase complexes, CRL and RING, target ABA receptors for degradation

CRL and RING E3 ligase complexes were recently demonstrated to target PYR/PYLs for degradation via the Ub‐26S proteasome pathway (Bueso *et al*., 2014; Irigoyen *et al*., 2014). De‐etiolated 1 (DET1)‐damaged DNA binding protein 1 (DDB1)‐associated 1 (DDA1) is a component of COP10‐DET1‐DDB1‐related E3 ligase complexes via an interaction with DDB1. DDB1 is an essential member of the cullin 4 (CUL4)/DDB1 E3 ligase complex; it acts as the substrate receptor in CUL4 E3 ligases. DDA1 physically associates with PYL8, PYL4 and PYL9. It has been demonstrated that DDA1 targets PYL8 for degradation through 26S proteasome mediated by ubiquitination (Irigoyen *et al*., 2014). The presence of ABA blocks the ubiquitination of PYR/PYLs and stabilizes them (Irigoyen *et al*., 2014). Distinct subcellular localization patterns of E3 ligase complexes involved in ABA signalling have been observed. DDA1 controls the stability of PYL8 through nuclear colocalization (Irigoyen *et al*., 2014).

Ring finger of seed longevity 1 (RSL1), a single‐subunit RING‐type E3 Ub ligase, interacts directly with the ABA receptors PYR1 and PYL4 (Bueso *et al*., 2014). RSL1 catalyses the polyubiquitination of PYR1 and PYL4 to promote their degradation via the 26S proteasome (Bueso *et al*., 2014). Interestingly, RNA interference (RNAi) mutants in which at least three members of the RSL1‐like gene family were silenced showed ABA‐hypersensitive phenotypes in terms of the ABA‐mediated inhibition of seed germination and early seedling growth, whereas RSL1 overexpression reduced ABA sensitivity (Bueso *et al*., 2014). These findings suggest that RSL1 acts as a negative regulator of ABA signalling by mediating the degradation of ABA receptors (Bueso *et al*., 2014). The ubiquitination of PYR1 or PYL4 catalysed by RSL1 occurs at the plasma membrane (Bueso *et al*., 2014). Ubiquitinated plasma membrane proteins are usually delivered to the vacuolar degradation pathway by the endosomal sorting complex required for transport (ESCRT) machinery (MacGurn *et al*., 2012). Recently, the PYL4 was identified to interact with FYVE 1 (also termed as FYVE domain protein required for endosomal sorting 1, FREE1), a FYVE domain‐containing protein (Belda‐Palazon *et al*., 2016). FYVE1 is the only ESCRT component that binds to ubiquitin and mediates vacuolar sorting of proteins (Gao *et al*., 2015; Kolb *et al*., 2015). Mutations of *fyve1* result in the accumulation of PYL4 and showed ABA‐hypersensitive phenotype in seed germination and root growth. The ubiquitylated PYL4 ABA receptor is the substrate for FYVE1 to mediate their delivery to the vacuolar degradation pathway (Belda‐Palazon *et al*., 2016). This is first discovery on the involvement of the ESCRT machinery in ABA signalling.

### PP2C and PP2A are substrates of plant U‐box (PUB) and C‐terminus of Hsc70‐interacting protein (CHIP) E3 ligases

Group A PP2Cs have been found to be substrates of U‐box E3 ligases and RING E3 ubiquitin ligases in ABA signalling (Kong *et al*., 2015). The PUB E3 ligases PUB12 and PUB13 specifically target ABI1, but not ABI2, for degradation. It has been shown that PYR1 interacts with ABI1 in an ABA‐dependent manner (Park *et al*., 2009). In an *in vitro* assay, ABI1 was ubiquitinated by PUB12 and PUB13 only in the presence of ABA and PYR1, indicating that ABA and PYR1 facilitate the ubiquitination of ABI1 (Kong *et al*., 2015). The double mutant *pub12 pub13* showed ABA insensitivity; however, introducing the *abi1‐3* loss‐of‐function mutation into that mutant recovered its ABA‐insensitive phenotype. The accumulation of ABI1 in *pub12 pub13* mutant plants has also been observed (Kong *et al*., 2015). These results suggest that ABI1 stability, controlled by PUB12 and PUB13, may contribute to the efficient release of ABI1 repression in the presence of ABA or under conditions of stress (Kong *et al*., 2015).

The ring domain ligase (RGLG) 1 and RGLG5 have also been demonstrated to be involved in ABA signalling, which belong to RING E3 ligases (Wu *et al*., 2016; Yin *et al*., 2007). They physically interact with PP2CA, ABI2 and HAB2, which is enhanced by ABA. RGLG1 and RGLG5 catalyse ubiquitination of PP2CA, possibly ABI2 and HAB2, and target them for degradation through 26S proteasome (Wu *et al*., 2016). Loss‐of‐function mutants of RGLG1 and RGLG5 showed ABA‐insensitive phenotypes in germination and postgermination growth. These results suggest that both RGLG1 and RGLG5 are positive regulators of ABA signalling through disrupting repressors of ABA signalling to activate ABA pathway (Wu *et al*., 2016).

CHIP is an E3 Ub ligase possessing monoubiquitination activity in *Arabidopsis*. Both RCN1 and PP2AA3 regulatory subunits of PP2A are substrates of AtCHIP (Luo *et al*., 2006). The overexpression of *AtCHIP* causes ABA hypersensitivity during seed germination and ABA‐induced stomatal closure (Luo *et al*., 2006). AtCHIP can monoubiquitinate PP2AA3 or RCN1 regulatory subunits of PP2A and enhance PP2A activity under stressful conditions, but it does not affect the enzyme's stability (Luo *et al*., 2006). However, the position and function of AtCHIP‐mediated PP2AA3 or RCN1 monoubiquitination must be resolved.

Although both PP2Cs and PP2As are regulated by ubiquitination, the regulatory mechanisms are different. PUB12 and PUB13 E3 ligases target ABI1 for degradation via the 26S proteasome (Kong *et al*., 2015). However, the CHIP E3 ligase monoubiquitinates PP2As, enhancing their phosphatase activity (Luo *et al*., 2006). These results suggest different roles for ubiquitination in the regulation of ABA signalling.

### CRL and RING E3 ligase complexes regulate ABA signalling by targeting transcription factors for turnover

Several transcription factors involved in ABA signalling (ABI5, ABF1, ABF3, ABI3, ATHB6, and salt‐ and drought‐induced RING finger 1 [SDIR1]‐interacting protein 1 [SDIRIP1]) can be ubiquitinated and degraded via Ub‐mediated proteolysis. Further, several specific E3 ligases have been discovered (Chen *et al*., 2013; Lee *et al*., 2010; Liu and Stone, 2013; Lyzenga *et al*., 2013; Seo *et al*., 2014; Stone *et al*., 2006).

DWD hypersensitive to ABA1 and ABA2 (DWA1 and DWA2, respectively) and ABA‐hypersensitive DCAF 1 (ABD1), the substrate receptors in CUL4‐RING E3 ligase complexes, are involved in ABA signalling (Lee *et al*., 2010). DWA1, DWA2 and ABD1 bind to ABI5 and promote its degradation by the 26S proteasome (Lee *et al*., 2010; Seo *et al*., 2014). Both *dwa1‐1/dwa2‐1* double mutant and *abd1* single mutant plants were found to be hypersensitive to ABA during seed germination and seedling growth and to exhibit retarded degradation of ABI5, indicating that DWA1, DWA2 and ABD1 are negative regulators of ABA signalling (Lee *et al*., 2010; Seo *et al*., 2014). In addition, ABI5 binding protein (AFP) has been identified as a negative regulator of ABI5 (Lopez‐Molina *et al*., 2003). AFP represses ABA signalling by promoting ABI5 degradation via the Ub‐mediated 26S proteasome pathway (Lopez‐Molina *et al*., 2003). However, whether AFP represses signalling by promoting ABI5 degradation is subject to debate. DWA1, DWA2, ABD1 and AFP are localized to the nucleus, where they target ABI5 for Ub‐mediated degradation (Lee *et al*., 2010; Lopez‐Molina *et al*., 2003; Seo *et al*., 2014).

Keep on going (KEG), a RING‐type E3 ligase, negatively regulates ABA signalling by targeting bZIP transcription factors, including ABI5, ABF1 and ABF3, and the kinase CIPK26 for Ub‐mediated degradation (Chen *et al*., 2013; Liu and Stone, 2013; Lyzenga *et al*., 2013; Stone *et al*., 2006). The ubiquitination activity of KEG has been demonstrated *in vitro* (Stone *et al*., 2006). A T‐DNA insertion mutant of *KEG* was shown to be extremely sensitive to ABA in the inhibition of root growth, suggesting that KEG is a negative regulator of ABA signalling (Lyzenga *et al*., 2013). KEG interacts directly with ABI5, ABF1 and ABF3, which are essential for ABA‐mediated responses. In the absence of ABA, KEG targets ABI5, ABF1 and ABF3 for degradation to maintain low levels of these proteins (Chen *et al*., 2013; Liu and Stone, 2013; Lyzenga *et al*., 2013; Stone *et al*., 2006). The presence of ABA increases the abundance of ABI5, ABF1 and ABF3 and positively regulates their stability (Chen *et al*., 2013; Lopez‐Molina *et al*., 2001). ABA promotes KEG self‐ubiquitination and degradation via Ub‐mediated proteolysis, resulting in the turnover of KEG and the accumulation of ABI5, ABF1 and ABF3 leading to downstream ABA responses (Chen *et al*., 2013; Liu and Stone, 2010). KEG is localized to the trans‐Golgi network/early endosome and interacts with ABI5. It is thought that KEG targets ABI5 for degradation in the cytoplasm or trans‐Golgi network before it reaches the nucleus (Liu and Stone, 2013). Moreover, KEG interacts physically with and ubiquitinates CIPK26, causing its degradation by the 26S proteasome. KEG overexpression enhances the turnover of CIPK26 (Lyzenga *et al*., 2013).

Similar to ABI5, the level of ABI3 is regulated by proteasomal degradation mediated by ABI3‐interacting protein 2 (AIP2), a C3H2C3‐type RING‐motif protein that functions as an E3 ligase. ABI3 is an unstable protein containing four functional domains: A1, B1, B2 and B3 (Kurup *et al*., 2000). AIP2 interacts physically with ABI3 and acts as a negative regulator of ABA signalling by targeting ABI3 for destruction through the 26S proteasome. The null mutant *aip2‐1* was shown to possess increased levels of ABI3 compared with wild type and to be hypersensitive to ABA. Further work revealed that exogenous or endogenous ABA facilitates the accumulation of *ABI3* mRNA and protein, and it triggers downstream ABA responses by directly or indirectly interrupting the interaction of AIP2 with ABI3 (Kurup *et al*., 2000).

As substrate‐binding adaptors of CUL3‐based Ub E3 ligases, meprin and TRAF homology‐BTB proteins (termed BPMs) target a class I homeobox‐leucine zipper transcription factor, ATHB6, for degradation (Lechner *et al*., 2011). ATHB6 functions as a negative regulator of ABA responses (Himmelbach *et al*., 2002). In *bpm* RNAi lines, the level of ATHB6 was increased while the ubiquitinated form of ATHB6 was decreased compared to wild type. However, overexpression of *BPM6* accelerated the turnover of ATHB6, suggesting that BPMs influence ATHB6 stability (Lechner *et al*., 2011). Further, *bpm* RNAi transgenic lines exhibited an ABA‐insensitive phenotype in stomatal closure, indicating that BPMs play a positive role in ABA signalling (Johannesson *et al*., 2003; Lechner *et al*., 2011).

SDIR1, a RING finger E3 ligase, is also involved in ABA signal transduction (Zhang *et al*., 2007, 2015). The expression of *SDIR1* is universal, especially in stomatal guard cells and leaf mesophyll cells under drought stress. The RING finger domain of SDIR1 is required for its E3 Ub ligase activity (Zhang *et al*., 2007). Overexpression of *SDIR1* leads to ABA‐hypersensitive phenotypes in seed germination and stomatal closure. In contrast, the *sdir1‐1* mutant exhibited an ABA‐insensitive phenotype (Zhang *et al*., 2007). The results of genetic studies show that SDIR1 acts upstream of bZIP family genes, including *ABI5*,* ABF3* and *ABF4*, and that it positively regulates ABA signalling (Zhang *et al*., 2007). SDIRIP1, a direct target of SDIR1, was recently identified using cell biological, molecular biological and biochemical approaches (Zhang *et al*., 2015). SDIR1 interacts with and ubiquitinates SDIRIP1, marking it for degradation by the 26S proteasome. SDIRIP1‐RNAi lines showed ABA‐hypersensitive phenotypes, indicating that SDIRIP1 acts as a negative regulator of ABA signalling. SDIRIP1 selectively regulates the expression of ABI5, rather than ABF3 or ABF4, to regulate ABA‐mediated seed germination and responses to salt (Zhang *et al*., 2015). Overall, the SDIR1/SDIRIP1 complex plays a vital role in ABA signalling via the ubiquitination pathway (Zhang *et al*., 2007, 2015).

### RPN10, a component of the 26S proteasome, regulates ABI5 stability

The selectivity of the Ub‐mediated 26S proteasome pathway is achieved not only at the level of ubiquitination, but also by the proteasome itself (Smalle and Vierstra, 2004). The 26S proteasome is an ATP‐dependent, self‐compartmentalized protease that can be divided into two particles: the 20S core protease (CP) and 19S regulatory particle (RP). The RP confers both ATP dependence and substrate specificity to the holoenzyme, especially with respect to those substrates bearing a polyubiquitin tag, by binding to each end of the CP (Smalle and Vierstra, 2004). RPN10 is a subunit of the RP in *Arabidopsis*. The mutant *rpn10‐1* showed increased ABA sensitivity and ABI5 accumulation, suggesting that RPN10 is essential for the degradation of ABI5 by the proteasome. The kinetics of ABI5 accumulation with and without ABA suggests that ABI5 is normally short‐lived. Although multiple mechanisms could explain this, the simplest explanation is that ABI5 turnover is achieved through the interaction of ABI5 with RPN10. This interaction may be direct or indirect, dependent or independent of previous ubiquitination events, and may involve adaptor proteins such as radiation sensitive (RAD) 23, the RAD23/ABI3 complex or DSK2 to promote the association of ABI5 with the protease. ABA may stabilize ABI5 by preventing this association. Clearly, identifying the proteins that interact with RPN10 will be critical in defining how it helps to deliver appropriate cargo to the 26S proteasome (Smalle *et al*., 2003).

### AtAIRP3/LOG2, a RING E3 ligase, targets *RESPONSIVE TO DEHYDRATION 21* (*RD21*), an ABA‐responsive gene, for disruption

Besides the aforementioned post‐translational regulation of upstream factors involved in ABA perception and signal transduction, downstream ABA‐responsive genes are subject to ubiquitination (Kim and Kim, 2013). AtAIRP3/LOG2, a RING‐type E3 ligase, is involved in ABA signalling. Both the knockout mutant *atairp3/log2‐2* and AtAIRP3‐RNAi knockdown transgenic plants exhibited ABA‐insensitive phenotypes in the inhibition of seed germination and stomatal closure, indicating a positive role for AIRP3 in ABA responses (Kim and Kim, 2013; Pratelli *et al*., 2012). In *Arabidopsis*, AtAIRP3/LOG2 interacts with RD21, a drought‐inducible cysteine proteinase belonging to the papain family (Kim and Kim, 2013). AIRP3 ubiquitinates RD21, targeting it for degradation by the 26S proteasome. These data indicate that AIPR3 functions as an E3 ligase towards RD21 and that it acts positively in ABA signalling (Kim and Kim, 2013).

## Perspectives

ABA plays important roles in regulating plant development and tolerance to biotic and abiotic stresses. As the characterization of PYR/PYLs as ABA receptors, great progress has been made in understanding the regulation (especially the post‐translational regulation) of ABA signalling. Dephosphorylation, phosphorylation and ubiquitination have all been reported to be involved in ABA signalling. After binding to ABA, PYR/PYLs interact physically with group A PP2Cs, disrupting the interaction between PP2Cs and SnRK2s, blocking the dephosphorylation activity of PP2Cs and activating SnRK2s to trigger downstream ABA responses. Multiple substrates of SnRK2s have been identified, including transcription factors, ion channel proteins, a potassium channel protein and a chromatin‐remodelling factor. The ubiquitination of ABA receptors and signalling molecules was recently reported, implying the importance of this modification in ABA signalling. However, certain problems must be addressed in order for us to fully understand the molecular networks involved in ABA signalling.

First, studies are needed to dissect the transcriptional, post‐transcriptional and post‐translational regulatory mechanisms of PYR/PYLs. Although it has been shown that PYL4, PYL8 and PYL9 are targeted by DDA1 for degradation by the 26S proteasome, PYR1 and PYL4 are substrates of RSL1, an E3 ubiquitin ligase; thus, the regulation of ABA receptors is far from clear (Bueso *et al*., 2014; Irigoyen *et al*., 2014). Second, even though autophosphorylation is a possible means of regulation for SnRK2s, the mechanisms underlying the internal phosphorylation of SnRK2 are obscure (Sirichandra *et al*., 2009b). In addition, other types of kinases, similar to SnRK2s and CIPK26, are likely involved in ABA signalling. Third, the targets of a great number of E3 Ub ligases involved in ABA signalling should be clarified in the future. Numerous E3 ligases, including DOR (Zhang *et al*., 2008), EID1‐like protein 3 (Koops *et al*., 2011), MAX2 (Bu *et al*., 2014), DWA3 (Lee *et al*., 2011), PUB9 (Samuel *et al*., 2008), PUB18 (Bergler and Hoth, 2011), PUB19 (Liu *et al*., 2011), AIRP1 (Ryu *et al*., 2010), AIRP2 (Cho *et al*., 2011), AIRP4 (Yang *et al*., 2016), ATL43 (Serrano *et al*., 2006), ECERIFERUM 9 (CER9) (Zhao *et al*., 2014), RDUF1/2 (Kim *et al*., 2012), RHA2a/2b (Bu *et al*., 2009; Li *et al*., 2011) and TLP3/9 (Bao *et al*., 2014), have been shown to play positive or negative roles in ABA signalling. However, their specificities are unknown.

The identification of their substrates will deepen our understanding of the mechanisms underlying ABA signalling networks.

## Conflict of Interest

There is no any conflict of interest being declared.

## Funding

This work was supported by National Natural Science Foundation of China (NSFC) [grant number 31371222] and Doctoral Fund of Ministry of Education of China (RFDP) [grant number 20132103110004].
